# Prevalence of generalized joint hypermobility, hypermobility spectrum disorders and Ehlers-Danlos syndrome: systematic review and meta-analysis

**DOI:** 10.1007/s00296-026-06346-3

**Published:** 2026-11-05

**Authors:** Ida Carnerup, Elisabeth Bondesson, Eric Ahl, Anna Trulsson Schouenborg, Anna Saxne Jöud

**Affiliations:** 1 Orthopedics, Department of Clinical Sciences Lund, Lund University, Remissgatan 4, Lund, 222 42 Sweden; 2 Department of Neurosurgery and Pain Rehabilitation, Skåne University Hospital, Lund, Sweden; 3 Department of Research, Education, Development and Innovation, HTA South, Skåne University Hospital, Lund, Sweden; 4 Department of Health Sciences, Research Group Human Movement - Health and Rehabilitation, Lund University, Lund, Sweden; 5 Occupational and Environmental medicine, Department of Laboratory medicine, Lund University, Lund, Sweden

**Keywords:** Ehlers Danlos syndrome, Hypermobility spectrum disorder, Generalized joint hypermobility, Systematic review, Prevalence, Epidemiology

## Abstract

Generalized joint hypermobility, GJH is common. Hypermobility can have various negative consequences and comorbidities, especially when criterion for Hypermobility Spectrum Disorder, HSD and Ehlers-Danlos syndrome, EDS are met.The prevalence of these different conditions is lacking. Therefore, the aim of this study was to assess the prevalence of these hypermobility disorders. A systematic search was conducted January 25, 2024 and updated May 28, 2025. It included the databases Ovid MEDLINE, Ovid Embase, CINAHL, and PEDro and followed a predefined search strategy adapted for each database (PROSPERO CRD42024505862) and included the MESH terms ‘Ehlers Danlos Syndrome’,‘Prevalence’, ‘Epidemiology’ and keywords ‘Hypermobility Spectrum Disorder’;‘Generalized joint hypermobility’ and ‘Systematic review’. 3633 unique records were screened, and 17 studies were included. From the meta-analysis the pooled prevalence across included studies of GJH in females were 9.5% (95% CI: 5.5; 15.8) with an I² of 95.5 % and 5.2% (95% CI: 3.0; 9.0) in males with an I² of 94.8). The evidence base for the age group 51 and older was insufficient for a robust estimate. Only one single study assessed HSD and hypermobile Ehlers-Danlos syndrome, hEDS in individuals aged 18-50 years and reported a prevalence of HSD of 38% in females and 39% in males, and the prevalence of hEDS as 2% in females and 0% in males. Hence, the available evidence did not allow for robust conclusions regarding the population-level prevalence of HSD/hEDS. This study shows that GJH is common, but with considerable heterogeneity and broad prediction intervals, which makes it crucial to interpret the pooled prevalence with caution. Moreover, a significant knowledge gap in the prevalence of GJH but particularly for HSD and EDS was identified. Our findings highlight the difficulties yet the need for more prevalence data on these conditions to understand the impact on the individual patient, health care services, and society.

**Supplementary Information:**

The online version contains supplementary material available at https://doi.org/10.1007/s00296-026-06346-3.

## Introduction

Generalized joint hypermobility, GJH is common [1] and can be verified through a physical examination. Previous studies have reported a prevalence ranging from between 2 and 57% depending on definition [2, 3]. For most affected individuals, there are few negative health effects, and GJH occurs as a stand-alone symptom. For some individuals, GJH may even be beneficial when pursuing certain sports [1]. However, hypermobility, generalized or not, can have adverse negative effects that require healthcare [4]. Complications related to hypermobility can include musculoskeletal pain, fatigue, headaches, anxiety, and abdominal and genitourinary complaints [5]. Hypermobility is a mandatory symptom in hypermobility spectrum disorder (HSD) [6]. HSD is a clinically assessed diagnosis, characterized by joint hypermobility and associated symptoms such as chronic pain, impaired proprioception, and other musculoskeletal manifestations. The specific underlying causes are poorly understood [4]. Apart from HSD, hypermobility can also occur in other conditions, such as in Ehlers-Danlos Syndrome (EDS). EDS is a heterogeneous group of connective tissue disorders, with shared characteristics such as hypermobility, skin hyperextensibility, and tissue fragility [7]. According to the 2017 international classification for the Ehlers-Danlos syndromes, 13 subtypes that have been defined, with varying symptoms and severity [7]. Twelve of these 13 subtypes are confirmed by molecular diagnostics while the most common one, hypermobile Ehlers-Danlos syndrome (hEDS), relies solely on a thorough clinical examination. As hEDS encompasses a broad spectrum of symptom presentations and severity levels, the diagnostic procedure can be challenging [7]. In both HSD and hEDS pain is a common, often widespread and persisting symptom [8]. Individuals with HSD and hEDS also report, besides the symptoms described for GJH, other symptoms, and comorbidities. These include a high rate of autonomic dysregulation, cardiovascular disorders, respiratory disorders and sleep disturbances [4, 9, 10]. Additionally, complaints like depression, bipolar disorder [11] and neurodevelopmental disorders [9, 11–13] are also reported. These comorbidities can further impact the individual´s function, daily activity, and quality of life [14, 15].

GJH maybe overlooked due to its heterogeneous clinical presentation, limited awareness among healthcare professionals, and the reliance on physical examination alone for diagnosis [5]. The combined prevalence of HSD and hEDS is estimated to be between 111 and 166 per 100 000 [16], indicating uncertainty in estimates. Currently, robust estimates of prevalence based on the present recommended classifications are warranted and may support resource allocation, treatment planning, and prevention of complications.

Therefore, the aim of this study was to assess the prevalence of GJH, HSD and EDS as confirmed by the 2017 international classification of the Ehlers-Danlos syndromes.

## Methods

This systematic review follows an a priori published PROSPERO protocol (registration number: CRD42024505862). It focusses on the prevalence of GJH, HSD and EDS stratified by sex and age.

### Population

Studies were eligible if they included prevalence data for the general population regardless of geographic area and involved individuals aged five [5] and above. Studies were excluded if addressed, for example a specific patient group or athletes, hence not targeting the general population.

### Condition

Conditions included were GJH, HSD and EDS as confirmed by the 2017 international classification of the Ehlers-Danlos syndromes [7] and *A framework for the classification of joint hypermobility and related conditions* [6]. Assessment of GJH required the use of Beighton score [17], as it is the most commonly used and reliable tool for the assessment of GJH [2, 6, 18]. Studies were considered applicable if they disclosed cutoff in accordance with current recommendations for adults [7] and for children [19]. As joint range of motion tends to decrease with age [20, 21], the Committee on behalf of the International Consortium on the Ehlers–Danlos syndromes, propose age-specific cutoffs for GJH; Beighton score ≥ 6 for pre-pubertal children and adolescents, ≥ 5 for pubertal males and females up to the age of 50, and ≥ 4 for those ≥ 51 years of age [7, 19]. Studies that used these cutoffs, or disclosed data that enabled calculation of prevalence for the different age groups were included. Studies that assessed historic hypermobility with the so called 5-part questionnaire (5PQ), were also considered applicable [22]. The prevalence estimates were presented by sex and by age groups 5–18, 18–50 and 51 or older.

### Type of studies

Observational studies were included. The studies needed to be accessible in full text and report original data of prevalence. Duplicate reports of studies, conference abstracts, letters, or studies that did not include original analyses were excluded. Studies investigating hypermobility in specific conditions/syndromes/injuries, clinical trials, case reports, narrative reviews, opinion pieces, comments, or editorials were excluded as well as articles with a study sample sizes of fewer than 100 participants an a pirori size limitation to minimize random estimates bias.

### Search strategy

The systematic search strategy followed the PRISMA guidelines [23] and was developed in consultation with an information specialist (author EA) and conducted from database inception up to January 25, 2024, in the databases Ovid MEDLINE, Ovid Embase, CINAHL, and PEDro. Only studies published in peer-reviewed journals were included, to ensure scientific review process was applied on included studies.

A predefined search strategy was used and adapted for each database with a combination of terms and text words including terms related to GJH, HSD, EDS and prevalence. An additional updated search was conducted on May 28, 2025. The detailed search syntax per database is presented in Supplementary Table S1 and Table S2.

### Study selection

Studies including children or adult populations (cross-sectional or cohort design), presenting prevalence of GJH, HSD or EDS were considered for inclusion. Citations identified from databases were checked for duplicates when uploaded to Covidence (Covidence systematic review software, Veritas Health Innovation, Melbourne, Australia. Available at www.covidence.org) Any remaining duplicates were removed manually during screening. Studies that reported the same population sample were considered duplications and only the study that included the largest sample of the overlapping samples was included. Titles and abstracts were screened by any two of the four reviewers. Based on the selection criteria, piloting of study selection was conducted by assessing four studies to ensure agreements among the reviewers. Throughout the selection process, disagreements were discussed among the reviewers until consensus was obtained. Studies perceived as eligible by two reviewers were included for full-text screening. The same screening procedure was then applied to full-text screening except that full consensus was needed for the study inclusion. The updated search was conducted in the same manner by two of the reviewers.

### Risk of bias assessment

Risk of bias was assessed using the Joanna Briggs Institute’s critical appraisal checklist for studies reporting prevalence data [24]. The risk of bias tool consists of 9 items addressing domains of bias plus assessments concerning external validity, internal validity, domain of measurement bias, and bias related to the analysis [25]. Its use has been recommended when assessing prevalence studies [26]. Four independent reviewers assessed the studies, in pairs of two. Studies were assessed based on whether the sample frame was appropriate to address the target population, the general population. It was also appraised whether the study subjects and the setting were described in detail and if data analysis was conducted with sufficient coverage of the identified sample. The conditions measurements had to be described and conducted in a standardized and reliable way, with adequate response rate and appropriate statistical analysis. Overall appraisal was also assessed qualitatively with particular attention to methodological limitations considered to be influencal. Throughout the assessment process, disagreements were discussed among the reviewers until consensus was obtained. The assessment was made with a systematical and critical appraisal, rather than based solely on the numerical summary of criteria met, according to recommendations. Studies perceived as eligible by a pair of reviewers were included.

### Data extraction

Data extraction was made by the four reviewers. Extracted data included: authors, year of publication, country, condition, study population size, cases and prevalence.

### Synthesis of results

Prevalence for the different age groups was calculated separately, due to different cutoffs for the various age groups (ages 5–18, 18–50, 51 or older) in the 2017 international classification of the Ehlers-Danlos syndromes. In the meta-analysis, a Generalized Linear Mixed Model was used to estimate the pooled prevalence. Subgroup analyses were performed for each age group, male/female and heterogeneity was estimated for both subgroups and the total pooled prevalence using Cochrane’s Q-test and the I^2^ index. The robustness of the pooled estimate was explored by conducting a leave-one-out sensitivity analysis where the effect each study has on the pooled estimate was explored. The meta-analysis was performed with R Statistical Software (v 4.3.3) using the “Meta” package (v 7.0–0) [27]. Meta-analysis was conducted of the respective conditions, separated by sex and age group, if two or more studies were identified reporting data for the subgroup. When stratified data was lacking or scarce, a narrative synthesis without meta-analysis (SWIM) was performed [28].

## Results

### Search results

**Fig. 1:**
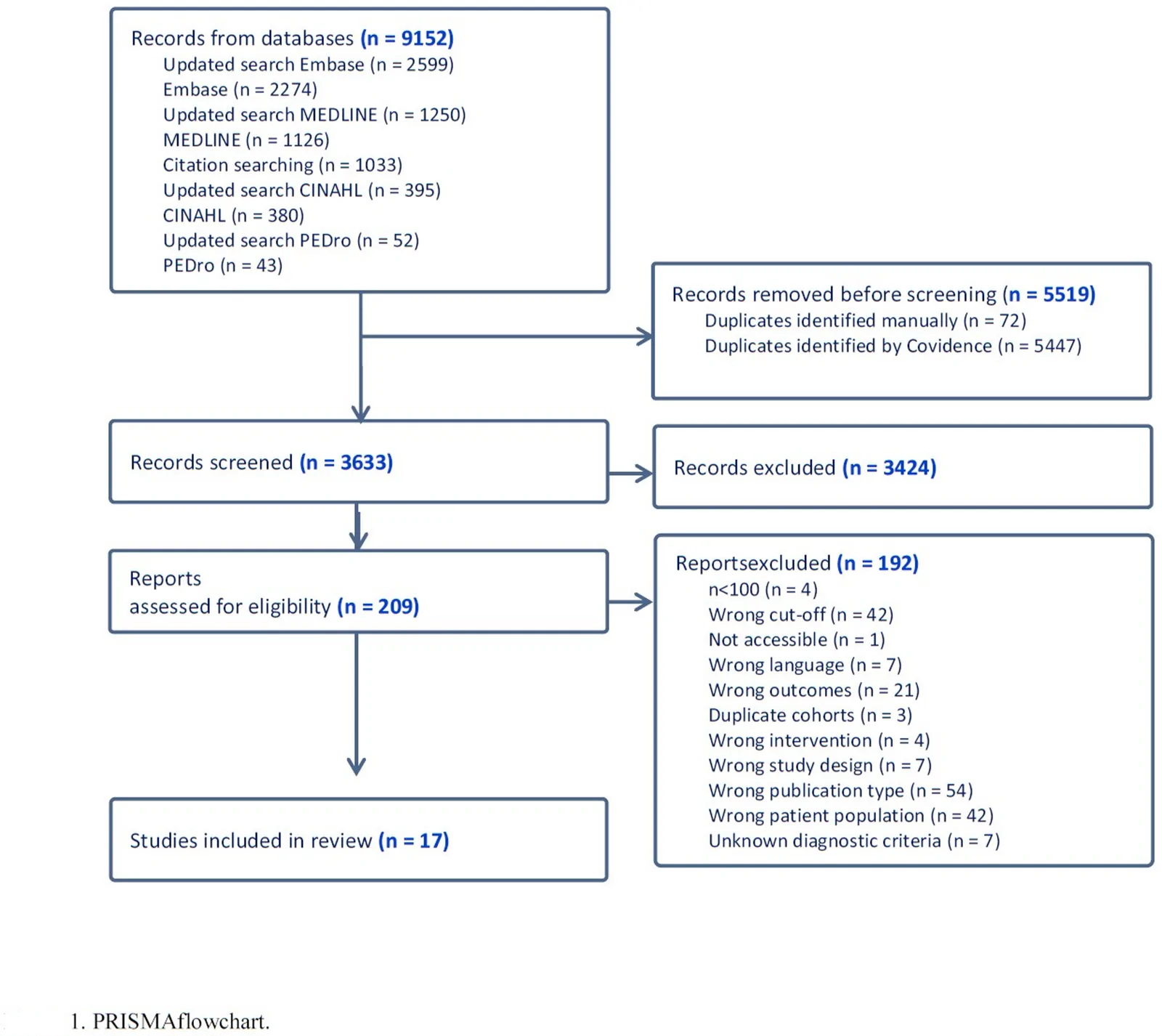
PRISMA flow chart

The search was conducted on January 25, 2024 and an updated search was conducted on May 28, 2025. A total of 9152 records were identified. After removing duplicates, 3633 unique records were screened. Title and abstract screening resulted in 209 studies reaching the full-text screening stage. A total of 17 studies met the inclusion criteria and were included after full text screening and Risk of Bias assessment (Fig. 1). None of the studies were excluded in the Risk of Bias-assessment (Table 1). The included study descriptives are presented in Table 2.

**Table 1:** Joanna Briggs Institute’s critical appraisal checklist, Risk of Bias-assessment

	1. Was the sample frame appropriate to address the target population?	2. Were study participants sampled in an appropriate way?	3. Was the sample size adequate?	4. Were the study subjects and the setting described in detail?	5. Was the data analysis conducted with sufficient coverage of the identified sample?	6. Were valid methods used for the identify-cation of the condition?	7. Was the condition measured in a standard, reliable way for all participants?	8. Was there appro-priate statistical analysis?	9. Was the response rate adequate, and if not, was the low response rate managed appropriately?	Overall appraisal
Butt 2014 [39]	Unclear	Unclear	Yes	Yes	Yes	Yes	Yes	Yes	Unclear	Include
Clinch 2011 [30]	Yes	Yes	Yes	Yes	Yes	Yes	Yes	Yes	Yes	Include
deBoer 2015 [31]	Yes	Unclear	Unclear	No	Unclear	Yes	Yes	Yes	Unclear	Include
Didia 2002 [44]	Yes	No	Yes	No	Unclear	Yes	Yes	Unclear	Unclear	Include
Dolan 2003 [38]	Yes	Yes	Yes	Yes	Yes	Yes	Yes	Yes	Yes	Include
Juul-Kristensen 2009 [32]	Yes	Yes	Yes	Yes	Yes	Yes	Yes	Yes	Yes	Include
Leone 2009 [33]	Yes	Unclear	Yes	Yes	Yes	Yes	Yes	Yes	Yes	Include
Mikkelsson 1996 [34]	Yes	Yes	Yes	Yes	Yes	Yes	Yes	Yes	Yes	Include
Morris 2017 [42]	Yes	Yes	Yes	Yes	Yes	Yes	Yes	Yes	Yes	Include
Qvindesland 1999 [35]	Yes	Unclear	Yes	Yes	Unclear	Yes	Yes	Yes	Unclear	Include
Remvig 2011 [36]	Yes	Yes	Yes	Unclear	Yes	Yes	Yes	Yes	Yes	Include
Reuter 2021 [45]	Unclear	Unclear	Yes	Yes	Yes	Unclear	Yes	Yes	Unclear	Include
Singh 2017 [43]	Yes	Unclear	Yes	No	Yes	Yes	Yes	Yes	Unclear	Include
Sirajudeen 2020 [40]	Yes	Yes	Yes	Yes	Yes	Yes	Yes	Yes	Yes	Include
Tuna 2020 [29]	Unclear	Yes	Yes	Yes	Yes	Yes	Yes	Yes	Yes	Include
Wincour 2000 [41]	Yes	Yes	Yes	Yes	Yes	Yes	Yes	Yes	Unclear	Include
Öhman 2014 [37]	Yes	Unclear	Yes	Yes	Yes	Yes	Yes	Yes	Unclear	Include

**Table 2:** Descriptives of included studies

References	Country	Type of population	Condition	Study population size	Cases	Prevalence
Butt 2014 [39]	Pakistan, Asia	School children	GJH	200 f 5-18 yrs 300 m 5- 18 yrs	67 f 87 m	33.5% 29%
Clinch 2011 [30]	United Kingdom, Europe	Routine health check-up at age 14	GJH	3061 f 5 -18 yrs 2961 m 5 - 18 yrs	214 f 39 m	7% 1.3%
deBoer 2015 [31]	The Netherlands, Europe	Child cohort study	GJH	138 f 5 -18 yrs 111 m 5 - 18 yrs	29 f 10 m	21% 9%
Didia 2002 [44]	Nigeria, Africa	University students	GJH	228 f 18- 50 yrs 192 m 18-50 yrs	31 f 11 m	13.6% 5.7%
Dolan 2003 [38]	UnitedKingdom, Europe	Population study	GJH	716 f > 51 yrs	1 f	0.1%
Juul-Kristensen 2009 [32]	Denmark, Europe	School children	GJH	160 f 5 - 18 yrs 189 m 5 - 18 yrs	15 f 18 m	9.4% 9.5%
Leone 2009 [33]	Italy, Europe	School children	GJH	516 f 5 - 18 yrs 530 m 5 - 18 yrs	91 f 67 m	17.6% 12.6%
Mikkelsson 1996 [34]	Finland, Europe	School children	GJH	835 f 5 - 18 yrs 802 m 5 - 18 yrs	70 f 57 m	8.4% 7.1%
Morris 2017 [42]	Australia	Pregnancy cohort (14 yrs)	GJH	815 f 5 - 18 yrs 769 m 5 - 18 yrs	94 f 201 m	26.1% 11.5%
Qvindesland 1999 [35]	Island, Europe	School children	GJH	143 f 5 - 18 yrs 124 m 5 - 18 yrs	20 f 6 m	14% 4.8%
Remvig 2011 [36]	Denmark, Europe	School children	GJH	159 f 5 - 18 yrs 156 m 5 - 18 yrs	19 f 16 m	12% 10.3%
Reuter 2021 [45]	USA, North America	University students	GJH	568 f 18 - 50 yrs 215 m 18 - 50 yrs	90 f 18 m	15.8% 8.4%
Singh 2017 [43]	Australia	Population study	GJH	102 f 5 - 18 yrs 102 m 5 - 18 yrs 100 f 18 - 50 yrs 100 m 18 -50 yrs 150 f ≥ 51 yrs 105 m > 51 yrs	6 f 0 m 6 f 1 m 0 f 0 m	5.9% 0% 6% 1% 0% 0%
Sirajudeen 2020 [40]	Saudi Arabia, Asia	School children	GJH	161 ≤ 18 yrs 142 m ≤ 18 yrs	16 f 7 m	10% 5%
Tuna 2020 [29]	Turkey, Europe	University students	GJH	221 f 18 - 50 yrs 114 m 18 -50 yrs	73 f 14 m	33% 12.3%
			HSD	221 f 18 - 50 yrs 114 m 18 -50 yrs	85 f 44 m	38.5% 38.6%
			hEDS	221 f 18 - 50 yrs 114 m 18 - 50 yrs	4 f 0 m	1.8% 0%
Wincour 2000 [41]	Israel, Asia	Junior high school	GJH	248 f 5 - 18 yrs	45 f	18%
Öhman 2014 [37]	Sweden, Europe	Elementary school	GJH	73 f 5 - 18 yrs 53 m ≤5 -18 yrs	4 f 0 m	9.6% 0%

GJH = Generalized joint hypermobility, HSD = Hypermobility spectrum disorder, hEDS=Hypermobile Ehlers-Danlos syndrome, f = female, m = male, yrs = years

### Study characteristics

**Fig. 2:**
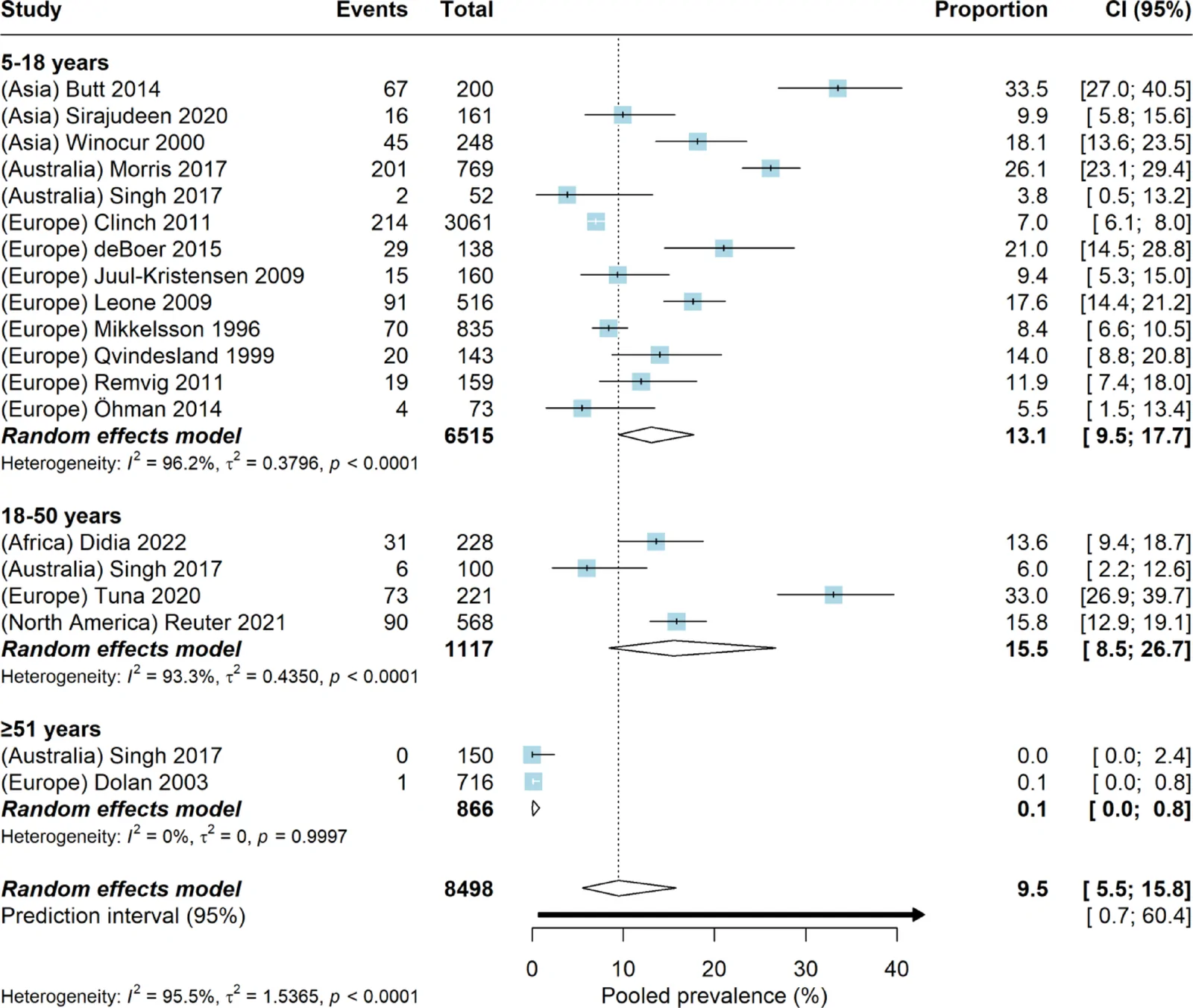
Prevalence of GJH in females

**Fig. 3:**
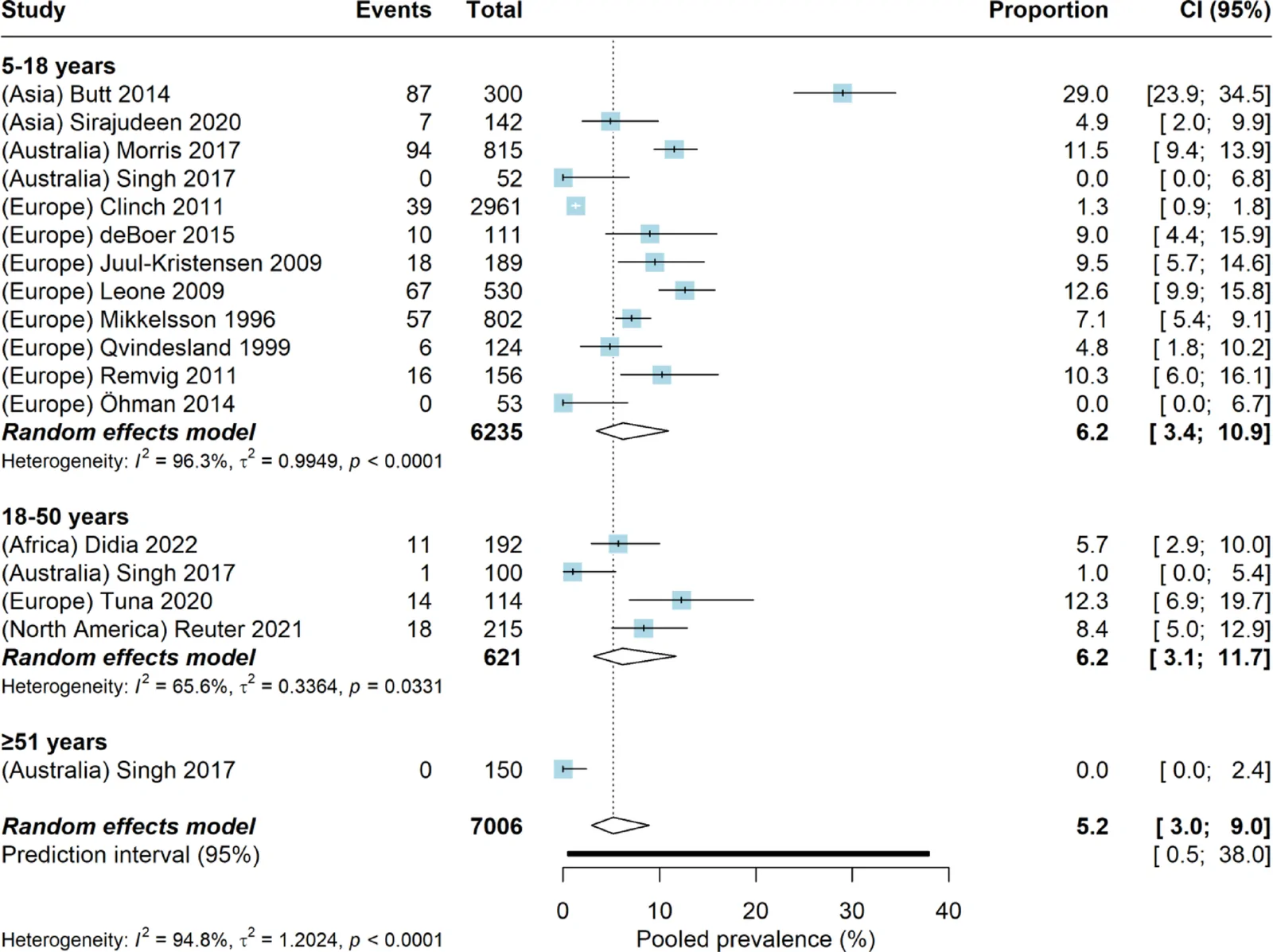
Prevalence of GJH in males

Out of the final 17 studies, three were from Asia, one from Africa, two from Australia, one from North America and 10 from Europe. All 17 studies covered GJH. Only one study reported prevalence of HSD and EDS, hence, only estimates for GJH could be pooled in the meta-analysis (Figs. 2 and 3) and no robust population-level prevalence conclusions regarding HSD/hEDS could be drawn. None of the studies reported the use of the 5PQ.

### Risk of bias assessment

In the risk of bias assessment (Table 1), there were primarily shortcomings identified in question 2, “Were study participants sampled in an appropriate way?” and 9. “Was the response rate adequate, and if not, was the low response rate managed appropriately?” with eight studies rated with shortcomings. Four studies had shortcomings identified in question 4. “Were the study subjects and the setting described in detail?”, and three in question 1. “Was the sample frame appropriate to address the target population?”, and in question 5. “Was the data analysis conducted with sufficient coverage of the identified sample?”. One study had shortcomings in 3. “Was the sample size adequate?”, 6. “Were valid methods used for the identify-cation of the condition?” and in question 8. “Was there appropriate statistical analysis?”

### Prevalence of GJH

The meta-analysis estimated the pooled prevalence across included studies of GJH to be 9.5% (95% CI: 5.5; 15.8) with an I² of 95.5% among females and 5.2% (95% CI: 3.0; 9.0) in males with an I² of 94.8%. Among girls aged 5–18 years, the prevalence was 13.1% (95% CI: 9.5; 17.7) with I² of 96.2% and in boys of the same age group it was 6.21% (95% CI: 3.4; 10.9 with an I² of 96.3%. In adult females and males (18–50 years) the pooled prevalence across included studies was 15.5% (95% CI: 8.5; 26.7) for females with an I² 93.3% and 6.2% (95% CI: 3.1; 11.7) for males with an I² of 65.6%. The leave-one-out sensitivity analysis did not change results (Supplementary figures S3-S8). In adults aged 51 or older the prevalence was reported in two studies for females and the pooled prevalence for this group was 0.1 (95% CI: 0.0; 0.8) with an I² of 0.0% and in males 0% (95% CI: 0.0; 2.4) (Figs. 2 and 3). The evidence base for the age group 51 and older was insufficient for a robust estimate.

### Prevalence of HSD and EDS

One study reported HSD in individuals aged 18–50 years, and they reported a prevalence of 38% in females and 39% in males [29]. This study was also the only one to assess the prevalence of hEDS in the 18–50 age group, finding 2% in females and 0% in males [29]. None of the studies covered HSD in the age groups 5–18 or 51 and older. Additionally, no studies reported data on the prevalence of any type of EDS in the age groups 5–18 or 51 or older. Other types of EDS besides hEDS were not reported in any group.

## Discussion

This systematic review and meta-analysis reveal an overall pooled prevalence across included studies of GJH of 9.5% (95% CI: 5.5; 15.8) with an I² of 95.5% among females and 5.2% (95% CI: 3.0; 9.0) in males with an I² of 94.8%, based on data from 17 studies. Due to heterogeneity between studies, it is crucial to interpret the result with caution. The I² of 95.5% among females and in males with an I² of 94.8% indicates considerable heterogeneity. These prediction intervals may provide a more clinically informative representation of the uncertainty than the pooled point estimates alone.

Only one study [29] could be identified that reported the subtypes of HSD and EDS, and this study only assessed prevalence in the age group 18–50. Hence, no robust estimates regarding the prevalence of HSD/hEDS could be made.

Regarding GJH, in the age group 5–18 years, the prevalence was 13.1%, (95% CI: 9.5; 17.7) in girls with an I² of 96.2% and in boys 6.2% (95% CI: 3.4; 10.9.) with an I² of 96.3% while the pooled prevalence for females and males aged 18–50 years was 15.5% (95% CI: 8.5; 26.7) with an I² of 93.3% for females and 6.2% (95% CI: 3.1; 11.7) with an I² of 65.6%. This difference in prevalence between the sexes were expected. The Beighton cutoff is age adjusted, and therefore the prevalence rates could be similar across different age groups. However, in individuals over the age of 51 or older, the evidence base was insufficient for a robust estimate. Only two studies reported prevalences in females aged 51 or older, and only one study reported prevalence for males aged 51 or older. This result supports the notion that GJH is a rare condition in higher age groups even though the cutoff is age-adjusted. Although, for this age group, the lack of data is an important limitation as only two studies reported prevalences in females aged 51 or older. For females 51 or older the pooled prevalence was 0.1% (CI: 0.0; 0.8) with a with an I² of 0%. This result should therefore be tempered as these estimates are based on very limited data as the evidence base for this age group was insufficient for a robust estimate.

The criteria for HSD and EDS can be used to diagnose biologically mature adolecents [6, 7] but are difficult to use in children and biologically immature adolecents [19]. A Beighton cut off 6/9 on Beighton score in children is advised [19]. As a quantitative assessment on a larger scale does not enable objective, individual maturity assessment age was set as a determinant.

The studies identified covering GJH included populations in Europe, Asia Australia and Africa. Ten studies assessed European populations [29–38], three Asian [39–41] ,two Australian populations [42, 43], one African [44] and one from North America [45]. Although somewhat unevenly distributed across continents, more assessments of prevalence in different ethnic populations are needed because of the genetic predisposition to GJH, especially EDS, associated to ethnicity [46]. We aimed to capture the general population; therefore, studies focusing on specific subgroups were excluded. At times, it was challenging to determine eligibility, but we strived to apply the inclusion criteria rigorously. In cases of uncertainty, consensus decisions were required. Notably, influence analyses did not meaningfully change the results as leave-one-out sensitivity analysis showed that the pooled prevalence estimate remained relatively stable when individual studies were sequentially excluded, indicating that the overall estimate was not driven by any single study. Rather substantial heterogeneity persisted across the analyses.

Only one of the included studies [29] reported prevalence of all of the three conditions GJH, HSD and hEDS. They found the prevalence of GJH to be 29% and the prevalence of HSD to be 38% in university students in Turkey. The prevalence for hEDS was estimated to 2% for females while there were no cases in males. Noteworthy, the prevalence of GJH among adult females in this same study was 33% and 12% in adult males. This can be compared to the pooled prevalence of GJH which was 9.5% for adult females and 5.2% for adult males in our meta-analysis. This large difference raises concerns about whether their results are generalizable to other populations, and the evidence base for robust estimates of prevalence of HSD and EDS are insufficient.

Prevalence of criteria-based conditions changes as criteria change. This systematic review presents the prevalence published with the criteria of the 2017 international classification of the Ehlers-Danlos syndromes. New criteria for HSD and EDS will be presented in the future, and this may alter the reported prevalence in the years to come. Therefore, this study provides important, systematically compiled data on prevalence based on the 2017 international classification criteria, allowing for future meaningful comparisons.

### Strengths and limitations

Initially, the search yielded a high number of articles. After screening only 17 articles were considered relevant. Many articles were excluded as the study population or the results of assessments weren´t described in sufficient detail, making it impossible to allow extraction of the data needed for this review. This led to a smaller number of articles being included, but this stringency was important to ensure trustworthy interpretation. The results reveal an important lack of data for several conditions and subgroups. This is a limitation when interpreting the findings; however, identifying these data gaps is in itself an important outcome of this review. Addressing these knowledge gaps in future research could significantly influence how clinicians and researchers interpret prevalence estimates. It could also have an impact on the allocation of appropriate resources, care, and treatment for these populations, thereby improving their quality of life. One of the strengths of this systematic review is that it was conducted close to the update of newer criteria, ensuring that a large proportion of the data was based on criteria valid within the covered timeframe. The decision to rigorously follow current classifications [6] led to the exclusion of otherwise relevant studies. However, as prevalence seems to vary with different criteria, this decision appears to be valid. One study [29] provided insight into how new criteria can alter prevalence, showing hEDS prevalence of 1,8% with current criteria, compared to 19% using older criteria in the same population [47]. This underscores the need to be rigorous with the criteria.

One possible weakness, as for many systematic reviews, is publication bias. This risk was limited by an extensive search for potentially relevant studies; however unpublished prevalence studies might still inflict our results. Considerable methodological variations were identified between included studies as well as heterogeneity in how results were presented. This, we believe, mirrors the challenges of assessing these conditions in clinical settings. As GJH, HSD and hEDS are all clinically confirmed conditions, it is not viable to ensure that these assessments have been performed consistently. Risk of Bias was assessed using the Joanna Briggs Institute’s critical appraisal checklist for studies reporting prevalence data [24] and the assessment was made with a systematical and critical appraisal, rather than being based solely on the numerical summary of criteria met. Studies perceived as eligible by a pair of reviewers were included. The critical appraisal identified recurring shortcomings related primarily to participant sampling and response rates. As these items were predominantly rated as “Unclear”, more comprehensive reporting of the study methods may have enabled a more definitive assessment. Furthermore, the majority of “No” ratings were attributable to Question 4, “Were the study subjects and the setting described in detail?”, reflecting inadequate reporting of study populations and settings.

One other possible limitation of this review is related to the process of grouping by age. The five-year limitation was set in accordance with the lower age limit for diagnosing GJH. The cutoff 18 years was used as it is considered more widely used. Individuals aged 18 were included in two age groups, to ensure that studies with different age limits were not excluded. This could theoretically have led to individuals being applicable to two groups. However, when the data was extracted, we were able to determine that this was not a problem.

In this systematic review prevalence was stratified by sex. This was decided as there is support for different prevalence rates between the sexes [15, 22, 34, 35]. Dividing the population into relevant groups makes the results comprehensible and provides insights into the current state of knowledge in these subgroups and is also more useful for interpretation in a clinical setting.

## Conclusion

This study suggests that GJH is common with a pooled prevalence of included studies of 9.5% (95% CI: 5.5; 15.8) with an I² of 95.5% among females and 5.2% (95% CI: 3.0; 9.0) in males with an I² of 94.8%. There was considerable heterogeneity among reported prevalences and broad prediction intervals, which makes it crucial to interpret the pooled prevalence with caution. The evidence base for robust estimates was insufficient in HSD, EDS and for age group above 50 in GJH. Moreover, a significant knowledge gap in the prevalence of GJH but particularly for HSD and EDS was identified. Our findings highlight the difficulties yet the need for more prevalence data on these conditions to understand the impact on the individual patient, health care services, and society.

## Supplementary Information

Below is the link to the electronic supplementary material.

Supplementary Material 1

Supplementary Material 2

Supplementary Material 3

Supplementary Material 4

Supplementary Material 5

Supplementary Material 6

Supplementary Material 7

## Declarations

### Generative artificial intelligence

No generative artificial intelligence (AI) or AI-assisted technologies were used in the preparation of this manuscript. No part of this work is copied, published elsewhere, or generated by AI. No external commercial editing support was used. All authors take full responsibility for the integrity and accuracy of all aspects of this work.
