# Supplementary material for: Prevalence of generalized joint hypermobility, hypermobility spectrum disorders and Ehlers-Danlos syndrome: systematic review and meta-analysis
Source: Rheumatol Int. 2026 Nov 5;46(11):306. doi: 10.1007/s00296-026-06346-3 (PMC13739981; doi:10.1007/s00296-026-06346-3)
Supplement: Supplementary file 1 [file 296_2026_6346_MOESM1_ESM.docx]

Table S1.

Medline through Ovid

| Ovid MEDLINE(R) ALL 1946 to January 25, 2024 | | | | |
| --- | --- | --- | --- | --- |
| # | Query | Hits | | |
| 1 | exp Ehlers-Danlos Syndrome/ | 3781 | | |
| 2 | (ehler* danlo*).ti,ab,kf. | 4509 | | |
| 3 | (hypermobil* adj3 (syndrom* or disorder* or joint$1 or symptom* or generalized or generalised)).ti,ab,kf. | 2488 | | |
| 4 | or/1–3 | 6581 | | |
| 5 | exp Morbidity/ or exp Ehlers-Danlos Syndrome/ep | 658002 | | |
| 6 | (morbidit* or prevalence or incidence or occurrence* or ((number* or amount* or volume* or quantit* or total$1 or count$1 or figure* or rate$1 or ratio$1 or frequenc*) adj3 (patient* or case* or incident* or individual$1 or population))).ti,ab,kf. | 3200894 | | |
| 7 | or/5–6 | 3365147 | | |
| 8 | 4 and 7 | 1120 | | |
| Embase via Ovid 1974 to 2024 January 25, 2024 | | | | |
| # | Query | | Hits | |
| 1 | exp Ehlers Danlos Syndrome/ | | 6649 | |
| 2 | (ehler* danlo*).ti,ab,kf. | | 5721 | |
| 3 | (hypermobil* adj3 (syndrom* or disorder* or joint$1 or symptom*) or beighton*).ti,ab,kf. | | 3857 | |
| 4 | or/1–3 | | 9806 | |
| 5 | Morbidity/ or exp Prevalence/ or exp Incidence/ or Patient Volume/ or Epidemiological Data/ or exp Ehlers Danlos syndrome/ep | | 2023812 | |
| 6 | (morbidit* or prevalence or incidence or occurrence* or ((number* or amount* or volume* or quantit* or total$1 or count$1 or figure* or rate$1 or ratio$1 or frequenc*) adj3 (patient* or case* or incident* or individual$1 or population))).ti,ab,kf. | | 4674023 | |
| 7 | or/5–6 | | 5130498 | |
| 8 | 4 and 7 | | 2267 | |
| CINAHL January 25, 2024 | | | | |
| # | Query | | | Hits |
| 1 | MH “Ehlers-Danlos Syndrome” | | | 764 |
| 2 | TI (ehler* danlo*) OR AB (ehler* danlo*) | | | 839 |
| 3 | TI (hypermobil* N3 (syndrom* OR disorder* OR joint* OR symptom*) OR beighton*) OR AB (hypermobil* N3 (syndrom* OR disorder* OR joint* OR symptom*) OR beighton*) | | | 1001 |
| 4 | S1 OR S2 OR S3 | | | 1712 |
| 5 | MH “Morbidity+” OR MH "Ehlers-Danlos Syndrome/EP" | | | 196,158 |
| 6 | TI (morbidit* OR prevalence OR incidence OR occurrence* OR ((number* OR amount* OR volume* OR quantit* OR total# OR count# OR figure# OR rate# OE ratio# OR frequenc*) N3 (patient* OR case* OR incident* OR individual# OR population))) OR AB (morbidit* OR prevalence OR incidence OR occurrence* OR ((number* OR amount* OR volume* OR quantit* OR total# OR count# OR figure# OR rate# OE ratio# OR frequenc*) N3 (patient* OR case* OR incident* OR individual# OR population))) | | | 749489 |
| 7 | S5 OR S6 | | | 809916 |
| 8 | S4 AND S7 | | | 392 |

| PEDro | | |
| --- | --- | --- |
| 1 | ehlers danlos | 7 |
| 2 | hypermob* | 35 |

Table S2.

| Ovid MEDLINE(R) ALL 1946 to May 28, 2025 | | | | | |
| --- | --- | --- | --- | --- | --- |
| # | Query | Hits | | | |
| 1 | exp Ehlers-Danlos Syndrome/ | 3961 | | | |
| 2 | (ehler* danlo*).ti,ab,kf. | 4884 | | | |
| 3 | (hypermobil* adj3 (syndrom* or disorder* or joint$1 or symptom* or generali#ed)).ti,ab,kf. | 2573 | | | |
| 4 | beighton*.ti,ab,kf. | 659 | | | |
| 5 | or/1-4 | 7134 | | | |
| 6 | exp Morbidity/ or exp Ehlers-Danlos Syndrome/ep | 697618 | | | |
| 7 | (morbidit* or prevalence or incidence or occurrence* or ((number* or amount* or volume* or quantit* or total$1 or count$1 or figure* or rate$1 or ratio$1 or frequenc*) adj3 (patient* or case* or incident* or individual$1 or population))).ti,ab,kf. | 3492306 | | | |
| 8 | or/6-7 | 3658156 | | | |
| 9 | 5 and 8 | 1250 | | | |
| Embase 1974 to 2025 May 28 | | | | | |
| # | Query | | Hits | | |
| 1 | exp Ehlers Danlos Syndrome/ | | 7475 | | |
| 2 | (ehler* danlo*).ti,ab,kf. | | 6312 | | |
| 3 | (hypermobil* adj3 (syndrom* or disorder* or joint$1 or symptom* or generali#ed)).ti,ab,kf. | | 3993 | | |
| 4 | beighton*.ti,ab,kf. | | 1170 | | |
| 5 | or/1-4 | | 10894 | | |
| 6 | Morbidity/ or exp Prevalence/ or exp Incidence/ or Patient Volume/ or exp Ehlers Danlos syndrome/ep | | 2237961 | | |
| 7 | (morbidit* or prevalence or incidence or occurrence* or ((number* or amount* or volume* or quantit* or total$1 or count$1 or figure* or rate$1 or ratio$1 or frequenc*) adj3 (patient* or case* or incident* or individual$1 or population))).ti,ab,kf. | | 5136143 | | |
| 8 | or/6-7 | | 5615726 | | |
| 9 | 5 and 8 | | 2599 | | |
| CINAHL – Search performed on May 28, 2025 – Search modes: Boolean/Phrase (Proximity) | | | | | |
| # | Query | | | Hits | |
| 1 | MH “Ehlers-Danlos Syndrome” | | | 817 | |
| 2 | TI (ehler* danlo*) OR AB (ehler* danlo*) | | | 905 | |
| 3 | TI (hypermobil* N2 (syndrom* OR disorder* OR joint* OR symptom* OR generali#ed)) OR AB (hypermobil* N2 (syndrom* OR disorder* OR joint* OR symptom* OR generali#ed)) | | | 934 | |
| 4 | TI (beighton*) OR AB (beighton*) | | | 320 | |
| 5 | S1 OR S2 OR S3 OR S4 | | | 1,831 | |
| 6 | MH “Morbidity+” OR MH "Ehlers-Danlos Syndrome/EP" | | | 199,892 | |
| 7 | TI (morbidit* OR prevalence OR incidence OR occurrence* OR ((number* OR amount* OR volume* OR quantit* OR total# OR count# OR figure# OR rate# OE ratio# OR frequenc*) N2 (patient* OR case* OR incident* OR individual# OR population))) OR AB (morbidit* OR prevalence OR incidence OR occurrence* OR ((number* OR amount* OR volume* OR quantit* OR total# OR count# OR figure# OR rate# OE ratio# OR frequenc*) N2 (patient* OR case* OR incident* OR individual# OR population))) | | | 740,163 | |
| 8 | S6 OR S7 | | | 801,880 | |
| 9 | S5 AND S8 | | | 395 | |
| PEDro – Search performed on May 28, 2025 | | | | | |
| 1 | ehlers danlos | | | | 11 |
| 2 | hypermob* | | | | 41 |
